# Canine exposure to vector-borne pathogens in a rural Brazilian landscape

**DOI:** 10.1007/s11259-026-11571-z

**Published:** 2026-10-08

**Authors:** Carlos Roberto Cruz Ubirajara Filho, Geovana Mergulhão da Silva, Maria de Lara Oliveira Lima, Lucia Oliveira Macedo, Leucio Câmara Alves, Gílcia Aparecida de Carvalho, Rafael Antonio Nascimento Ramos

**Affiliations:** 1 Laboratory of Parasitology, Federal University of the Agreste of Pernambuco, Garanhuns, PE 55292-278 Brazil; 2 Department of Veterinary Medicine, Federal Rural University of Pernambuco, Recife, PE 52171-900 Brazil; 3 Department of Biology, Federal Rural University of Pernambuco, Recife, PE 52171-900 Brazil; 4 Department of Veterinary Pathobiology, College of Veterinary Medicine and Biological Sciences, Texas A&M University, College Station, TX 77843 USA

**Keywords:** American Trypanosomiasis, Visceral Leishmaniasis, Dogs, Antibodies, One Health

## Abstract

Vector-borne pathogens (VBPs) of medical and veterinary concern have mostly been studied in urban dog populations, while animals in rural settings, with limited access to ectoparasite prevention, have received less attention. In this study, we assessed canine exposure to VBPs in a rural Brazilian landscape. From August to October 2024, we collected 155 blood samples from dogs domiciled in a rural area of the municipality of Garanhuns, Pernambuco, Northeastern Brazil. Samples were assessed using an immunofluorescence antibody test (IFAT) to detect antibodies against *Trypanosoma cruzi* and using a rapid immunochromatographic test combined with an ELISA to detect *Leishmania infantum* antibodies. In addition, each sample was tested using a qualitative ELISA to detect *D. immitis* antigens and antibodies against *Anaplasma phagocytophilum*/*Anaplasma platys*, *Ehrlichia canis*/*Ehrlichia ewingii*, and *Borrelia burgdorferi* sensu lato. A total of 56.1% (87/155) of samples were reactive to at least one pathogen. Specifically, in 5.1% (8/155) were detected anti-*T. cruzi* antibodies, and in four of these samples (2.5%, 4/155) were co-detected antibodies anti-*L. infantum*. Antibodies against *Ehrlichia* spp. were observed in 50.9% (79/155), *Anaplasma* spp. in 8.3% (13/155), and *B. burgdorferi* in 0.6% (1/155). Antigens for *D. immitis* were detected in 1.2% (2/155). No statistical associations were observed on variables (sex, mobility, overnight stay and presence of ectoparasites) and pathogen seropositivity. In conclusion, dogs from rural areas are exposed to VBPs, which requires the use of ectoparasiticide preventive compounds to avoid ectoparasite infestation and reduce the risk of pathogen exposure.

## Introduction

Vector-borne pathogens (VBPs) affecting dogs include a diverse range of causative agents, represented primarily by bacteria (e.g., *Ehrlichia* spp., *Anaplasma* spp., *Rickettsia* spp., *Borrelia* spp.), protozoa (e.g., *Hepatozoon* spp., *Babesia* spp., *Leishmania* spp., *Trypanosoma cruzi*), and nematodes (e.g., *Dirofilaria* spp., *Acanthocheilonema* spp.) (Ramos et al. 2010, 2022; Macedo et al. 2022). These pathogens have a global distribution, with a higher prevalence in tropical regions where year-round vector exposure is common (Rocha et al. 2024).

These pathogens have been studied primarily in urban dog populations that routinely receive preventive products, whereas studies involving rural dogs with limited access to such measures remain scarce. In rural settings of tropical regions, limited ectoparasite prevention, combined with the presence of vectors year-round, and contact of domestic dogs and wild animals commonly found in forested environments, increases the risk of exposure to both well-known and lesser-known VBPs circulating in natural ecosystems.

Extending this focus to Brazil, *Leishmania* spp. and *T. cruzi* are among the most significant pathogens affecting both humans and domestic dogs, particularly in rural areas (Pavan et al. 2025). Both parasites may occur in sympatry in the same area, posing an additional challenge to the epidemiological understanding of these infections.

Besides these two zoonotic parasites (*Leishmania* spp. and *T. cruzi*), other VBPs warrant consideration. Tick-borne bacteria (*Ehrlichia* spp. and *Anaplasma* spp.) and the mosquito-borne parasite (*D. immitis*) are well documented in Brazil (Macedo et al. 2022; Ramos et al. 2022). *Ehrlichia* spp. and *Anaplasma* spp., transmitted by *Rhipicephalus sanguineus* sensu lato ticks, cause a disease marked by diverse clinical signs, including fever, bleeding, hemoglobinuria, petechiae, jaundice, death, and hematological abnormalities such as anemia and thrombocytopenia. In contrast, *D. immitis* causes heartworm disease, the leading cause of acquired cardiomyopathy in dogs worldwide (Esteves-Guimarães et al. 2024). In the present study, we assessed the exposure to the main VBPs of dogs in a rural landscape, in the Northeastern region of Brazil.

## Materials and methods

### Study area and ethical aspects

Animals assessed in this study were from a rural community of the municipality of Garanhuns (08°53′25″ S, 36°29′34″ W), located in the Agreste region of the state of Pernambuco, Northeastern Brazil. The region is at an altitude of 896 m above sea level, it is part of the Caatinga biome and presents typical characteristics of highland wetlands, with remnants of the Atlantic Forest. The mean annual temperature is approximately 21.7 °C, with annual precipitation ranging from 660 to 890 mm. Rainfall is seasonal, concentrated mainly between April and July, while relative humidity remains high throughout the year.

### Sampling and laboratory procedures

The minimum sample size was estimated at 150 animals, based on the previously established ratio of 1 dog per 4.4 humans, as described by Canatto et al. (2012). The human population of the study area was estimated at 2,100 inhabitants (IBGE, 2025), while the canine population was estimated at 477 animals. The required sample size was calculated using the StatCalc utility in Epi Info™ (version 7.2), based on the expected prevalence and a 95% confidence level (Thrusfield 2007). To ensure full geographic coverage, sampling points were randomly selected within the study area, with one animal sampled per household. Dogs were enrolled in the study after their owners provided written informed consent.

From August to October 2024, blood (*n* = 5 mL) was drawn from the cephalic veins of 155 domiciled dogs and kept in tubes with (Ethylenediaminetetraacetic acid - EDTA) and without anticoagulant. After centrifugation (2,000 g for 10 min) of tubes with no anticoagulant and separation of serum, samples were stored at -20 °C until serological analysis. Data on sex, mobility, overnight stay, and presence of ectoparasites were recorded.

The *T. cruzi* antibody detection was performed using the Immunofluorescence Antibody Test (IFAT) according the protocol described by Camargo (1966). Samples were considered positive when the entire peripheral surface of the antigen showed fluorescence at the at a cut-off ≥ 1:40. Readings were performed in an epifluorescent microscope (OLYMPUS BX60-FLA, Westborough, MA, United States). On each slide, positive (reactive serum of a dog from endemic area) and negative control (non-reactive serum of a dog from non-endemic area) were added. For this assay were used total *T. cruzi* antigens provided by the Laboratory of Biology of Tripanosomatids of the Oswaldo Cruz Foundation, Rio de Janeiro, Brazil.

For the detection of antibodies anti-*Leishmania infantum*, serum samples were initially screened using a rapid immunochromatographic test (TR-DPP^®^, Bio-Manguinhos-Fiocruz, Rio de Janeiro, RJ, Brazil), according to the manufacturer’s instructions. Afterwards, reactive samples were tested using an indirect enzyme-linked immunosorbent assay (ELISA/S7^®^, Biogene, Recife, PE, Brazil) following manufacturer’s recommendations. All samples were tested in duplicate, including blank, negative, and positive controls. Samples were considered positive when scored reactive simultaneously for both assays.

Samples were also individually tested using a qualitative ELISA (SNAP^®^ 4Dx^®^ Plus, IDEXX Laboratory, Westbrook, ME, United States), according to the manufacturer’s instructions. The test can detect *D. immitis* antigens and antibodies against *Anaplasma phagocytophilum*/*Anaplasma platys*, *Ehrlichia canis*/*Ehrlichia ewingii*, and *Borrelia burgdorferi* sensu lato.

### Data analysis

The prevalence of seroreactive and positive animals was calculated using the Wilson method, considering a 95% confidence interval (https://epitools.ausvet.com.au/ciproportion). To compare the frequency of positivity across groups (sex, mobility, overnight stay and presence of ectoparasites), the Chi-square test (χ²) with Yates’ correction was used; the adopted significance level was 5%. All analyses were performed using BioEstat software version 5.0.

## Results

A total of 56.1% (*n* = 87; 95% CI = 48.2–63.7) of samples were reactive to at least one pathogen. Specifically, 5.1% (*n* = 8; 95% CI = 2.6–9.8) presented anti-*T. cruzi* antibodies, and in 2.5% (*n* = 4; 95% CI = 1.0–6.4) of these samples, antibodies anti-*L. infantum* were co-detected. Of the seven samples that screened positive for *L. infantum* antibodies, four were confirmed by ELISA. Only these four dual-positive samples were used to calculate seroprevalence.

Antibodies anti-*Ehrlichia* spp., *Anaplasma* spp., and *B. burgdorferi* were detected in 50.9% (*n* = 79; 95% CI = 43.1–58.2), 8.3% (*n* = 13; 95% CI = 4.9–13.8), and 0.6% (*n* = 1; 95% CI = 0.1–3.5) of the samples, respectively. Antigens for *D. immitis* were detected in 1.2% (*n* = 2; 95% CI = 0.3–4.5) of the evaluated animals.

A total of 10.9% (*n* = 17; 95% CI = 6.9–16.8) presented co-infections by two or three pathogens (Table 1). The seroprevalence of the investigated pathogens according to sex, mobility, presence of ectoparasites, and overnight stay is presented in the Table 2. Overall, no statistically significant associations were observed between the evaluated variables and positivity (*p* > 0.05). Due to the low number, positive samples for *B. burgdorferi* (*n* = 1) and *D. immitis* (*n* = 2) were excluded from the Table 2. Because ticks were the only ectoparasites found during the physical examination, they will be referred to directly throughout the remainder of this text.

**Table 1:** Single and simultaneous positivity by canine vector-borne pathogens in animals residing in a rural landscape

Pathogen	Prevalence % (*n*)	95% CI
*Leishmania infantum*	1.94 (3)	0.66–5.54
*Ehrlichia canis*	40.00 (63)	32.62–7.86
*Anaplasma* spp.	0.65 (1)	0.11–3.56
*Trypanosoma cruzi*	1.29 (2)	0.35–4.58
*Dirofilaria immitis	1.29 (2)	0.35–4.58
*Leishmania infantum* + *Ehrlichia canis*	0.65 (1)	0.11–3.56
*Ehrlichia canis* + *Anaplasma* spp.	7.10 (11)	4.01–12.26
*Ehrlichia canis* + *Trypanosoma cruzi*	2.58 (4)	1.01–6.45
*Anaplasma* spp. + *Borrelia burgdorferi* + *Trypanosoma cruzi*	0.65 (1)	0.11–3.56

*Except for *D. immitis*, the results of all pathogens represent the serological prevalence

**Table 2:** Absolute and relative frequency of seroreactive dogs for *Leishmania infantum*, *Ehrlichia canis*,* Anaplasma* spp. and *Trypanosoma cruzi*, according to sex, mobility, presence of ticks and overnight stay

Variables	Leishmania infantum *n*/*N* (%; 95% CI)	χ2; *p* value	Ehrlichia canis *n*/*N* (%; 95% CI)	χ2; *p* value	Anaplasma spp. *n*/*N* (%; 95% CI)	χ2; *p* value	Trypanosoma cruzi *n*/*N* (%; 95% CI)	χ2 *p* value
Sex								
Male	3/86 (3.4%; 1.1–9.7)	0.0818; *p* = 0.77	39/86 (45.3%; 35.2–55.8)	1.9617; *p* = 0.16	5/86 (5.8%; 2.5–12.9)	0.9974; *p* = 0.32	4/86 (4.6%; 1.8–11.3)	0.002; *p* = 0.96
Female	1/69 (1.4%; 0.2–7.7)		40/69 (57.9%; 46.2–68.8)		8/69 (11.5%; 5.9–21.2)		4/69 (5.8%; 2.2–13.9)	
Mobility								
Semi-restricted	4/147 (2.7%; 1.0–6.8)	NP	74/147 (50.6%; 42.6–58 − 6)	0.0942; *p* = 0.75	13/147 (8.9%; 5.2–14.6)	NP	8/147 (5.4%; 2.8–10.4)	NP
Unrestricted	0/8 (0)		5/8 (62.5%; 30.5–86.3)		0/8 (0)		0/8 (0)	
Ticks								
Yes	1/35 (2.8%; 0.5–14.5)	0.2387; *p* = 0.23	17/35 (48.5%; 32.9–64.4)	0.0169; *p* = 0.89	3/35 (8.5%; 2.9–22.3)	0.0911; *p* = 0.76	1/35 (2.8%; 0.5–14.5)	0.0708; *p* = 0.79
Not	3/120 (2.5%; 0.8–7.0)		62/120 (51.6%; 42.8–60.4)		10/120 (8.3%; 4.5–14.6)		7/120 (5.8%; 2.8–11.5)	
Overnight stay								
Peridomicile	4/139 (2.8%; 1.1–7.1)	NP	71/139 (51.0%; 42.8–59.2)	0.0332; *p* = 0.85	11/139 (7.9%; 4.4–13.6)	0.0227; *p* = 0.88	7/139 (5.0%; 2.4–10.0)	0.1511; *p* = 0.69
Intradomicile	0/16 (0)		8/16 (50.0%; 28.0–72.0)		2/16 (12.5%; 35.0–36.0)		1/16 (6.2%; 1.1–28.3)	

*CI* confidence interval, *n *number of positive dogs, *N *number total of dogs. *χ2*  Chi -square. Significant at *p* < 0.05. NP indicates not possible to calculate

## Discussion

Findings from the present study indicates that dogs living in rural areas were exposed to a wide plethora of VBPs, some of them critical agents of One Health concern. The detection of antibodies against *T. cruzi*, *L. infantum*, *Anaplasma* spp., *E. canis*, *B. burgdorferi* and *D. immitis* antigens underscores the diversity of pathogens threatening this animal population. The substantial overall seroprevalence of 56.1% (87/155) suggests the lack of an effective arthropod control, through the use ectoparasiticide compounds. These findings align with recent reports of high prevalence rates for VBPs in rural-peri-urban transition zones, where limited sanitary infrastructure and favorable climate sustain vector populations throughout the year (Silva et al. 2023).

*Trypanosoma cruzi* seroprevalence (5.1%) matches with rates detected in states nearby the study area such as Alagoas (3.8%) and Paraíba (4.0%) (Oliveira Neto et al. 2026), but differs sharply from the states of Ceará (22%) and Piauí (71%). Even if in the present study, no vectors have been assessed, most likely these differences may be attributed to local climate influencing triatomine population and consequently dog exposure to *T. cruzi* parasites (Oliveira Neto et al. 2026). Although no statistically significant association was observed between epidemiological variables and positivity, exclusive detection in semi-restricted dogs and predominantly in animals living in the peridomicile reinforces triatomines adaptability to colonize peridomicile areas (Silva et al. 2024), underscoring the role of the dog as a domestic reservoir.

The seropositivity rate for *L. infantum* (2.5%) is consistent with the prevalence rate previously detected in which 2.4% of animals presented antibodies (Lins et al. 2018), but much lower than 16% of a study conducted nearly two decades prior (Santos et al. 2010). Despite these disparities may result from several factors such as changes in transmission dynamics, or improved control and diagnostic methods, in these studies the use of different serological techniques might be the cause of the high positivity rate of the prior study compared with more recent studies. In the study area, an autochthonous case of canine visceral leishmaniasis has been reported (Ubirajara Filho et al. 2021), and human visceral and cutaneous leishmaniasis have been notified by surveillance health agencies (Ubirajara Filho et al. 2023). The higher frequency in males compared with females, and in semi-restricted animals with access to peridomicile areas overnight, suggest a higher exposure to vectors. Interestingly, entomological surveys carried out in the study area revealed the presence of a single sandfly species (i.e., *Lutzomyia evandroi*) (Ubirajara Filho et al. 2020), which suggest the potential role of this species as vector. This hypothesis should be further tested.

Antibodies against both *T. cruzi* and *L. infantum* were co-detected in four animals. Cross-reactivity between animals exposed to these two pathogens is well-documented in endemic areas (Zanette et al. 2014). Due to the limitations of the serological assays used here, it is impossible to determine whether these four animals experienced a true dual infection or if the results stem from cross-reactivity. Diagnostic methods that directly detect the causative agents (e.g., molecular assays or microscopic analysis) are needed to confirm true infection status.

The substantial seropositivity for *E. canis* (50.9%) and *Anaplasma* spp. (8.3%) indicates that this dog population is highly exposed to *R. sanguineus* s.l. (Araújo et al. 2026). The co-detection of antibodies for both pathogens underscore the clinical and epidemiological complexity posed by exposure to a single vector (Perera et al. 2025). The diagnostic test employed does not confirm active infection, but indicates the presence of antibodies, which for some pathogens as *E. canis* may persist for months after infection (Evaristo et al. 2024). The unique detection of antibodies anti-*B. burgdorferi* in a single animal (0.6%) corroborates with a previous detection in the same study area (Macedo et al. 2022).

The diagnose of *D. immitis* antigen in two animals (1.2%) confirm the circulation of this nematode in this area of high altitude as previously reported (Macedo et al. 2022). Studies across seven coastal cities in the state Pernambuco showed a 3.6% canine infection rate which affirm the nematode persistence in different areas of the region (Rocha et al. 2024). The study area is located in a region of high altitude (896 asl), in which the unique microclimate, including seasonal wet periods with average temperatures slightly below 20 °C (May–August), reduces culicid population, justifying low local serological prevalence.

The absence of clinical data on animals, and no collection, identification and assessment of ticks detected at sampling, are important limitations of this study. Besides that, the diagnostic technique used for almost all pathogens (except for *D. immitis*), did not allow the determination of active infection, only pathogen exposure. Despite of limitation, the pronounced overall seroprevalence of VBPs observed in this study might has significant clinical consequences for dogs. These findings are critical for One Health, as dogs serve as reservoir of pathogens causing zoonoses. Strategic vector control is needed, emphasizing the One Health framework, with public health education, entomological surveillance and use of ectoparasiticide compounds year-round. In conclusion, dogs of this area are exposed to VBPs, and given their close human cohabitation, they may act as important epidemiological sentinels whose infection patterns mirror for some pathogens as *Leishmania* and *Trypanosoma* the zoonotic risk.

## Data Availability

No datasets were generated or analysed during the current study.
